# Composites prepared from the waterborne polyurethane cationomers-modified graphene. Part II. Electrical properties of the polyurethane films

**DOI:** 10.1007/s00396-015-3697-2

**Published:** 2015-07-15

**Authors:** Piotr Król, Bożena Król, Marek Zenker, Jan Subocz

**Affiliations:** Department of Polymer Science, Faculty of Chemistry, Rzeszów University of Technology, Al. Powstańców Warszawy 6, 35-959 Rzeszów, Poland; Faculty of Electrical Engineering, Department of Electrotechnology and Diagnostic, West Pomeranian University of Technology, Szczecin, Ul. Sikorskiego 37, 70-313 Szczecin, Poland

**Keywords:** Volume resistivity, Havriliak–Negami equation, Dielectric constants *ε*′ and *ε*″ in frequency domain, Dielectric losses coefficient tgδ

## Abstract

The research was planned to test electrical properties of polymer films made from polyurethane cationomers with 0–2 wt.% graphene admixture. The cationomers were synthetized in the reaction of 4,4′-methylenebis(phenyl isocyanate), polycaprolactone diol (*M* = 2000), *N*-methyldiethanolamine, and formic acid. It was found that addition of approx. 2 wt.% of graphene causes the loss of volume resistivity by three orders of magnitude and percolation threshold is already set at approx. 1 wt.%. The frequency characteristic of a real part of permittivity *ε*′ and imaginary part of permittivity *ε*″ were measured for the tested films. On the base of Havriliak–Negami equation, parameters of relaxation functions in frequency domain were estimated for samples containing various contents of graphene. The influence of the cationomer phase structure on observed changes of dielectric losses coefficient tgδ in the full-measuring frequency spectrum was discussed.

## Introduction

In the part I of the paper [1] synthesis, physicomechanical and thermal properties of films made of polyurethane cationomers modified with graphene in the amount of 0.5–2 wt.% were described. In order to allow good dispersion of graphene nanoparticles in polyurethane (PU), in the second stage of the polyaddition process, a suspension of graphene in THF was introduced to the resulting mixture of MDI and isocyanate prepolymer. Appropriate suspensions (with calculated quantity of graphene (0.024–0.484 g) in THF (7.5 cm^3^) were obtained in the process of sonication at 50 °C for 30 min in ELMASONIC P ultrasonic bath at a frequency of 80 kHz. It was found that the presence of graphene results in increased thermal and mechanical strength of received polymer films and contributes to the increase in hydrophobicity of generally hydrophilic coatings prepared from waterborne polyurethane cationomers [1]. Meanwhile, this paper is focused on the tests of electric properties of obtained films, expecting that presence of graphene particles, contrary to frequently used in polymer nanocomposites graphene oxide (GO), would result in lowering electrical resistivity of films and would lead to obtain materials with nonlinear dielectric properties, which might be used in electronics.

So far, there have been many descriptions of modifications of polymer electric properties, for typical dielectrics after admixture introduction of metal nanoparticles like Au, Ag, polyaniline, carbon black, or specially prepared multi-walled carbon nanotubes (MWCNT) [2–4]. Limiting the discussion to polyurethanes, which are of interest to this paper, it is worth noticing that there is the possibility of electric and magnetic properties changes of waterborne polyurethane nanocomposite based on anionomer polyurethane after introduction of Fe_3_O_4_ particles modified by oleic acid, which were dispersed in PPG glycol under ultrasonic bath. The addition of 0–3.5 wt.% of Fe_3_O_4_ caused the loss of the volume resistivity of obtained polymer foils from the value of 1.04 × 10^14^ to 5.96 × 10^4^ Ω cm and surface resistivity from the value 10^13^ to 10^5^ Ω cm^−2^ [5]. On the other hand, the presence of GO initially modified with 4,4′-MDI added in the amount of 1.5 to 3 % to thermoplastic polyurethane synthesized from MDI, poly(tetramethylene ether) glycol, and 1,4-butanediol had no influence on the conductivity; however, it resulted in a significant increase of dielectric constant (*ε*′) from the value of approx. 8 to 50 and dielectric loss constant (*ε*″) from the value of 0.1 to 0.9 measured by the frequency of 0.01 Hz. The measurements conducted in conditions of slow increase of frequency up to 10^6^ Hz in the case of these nanocomposites containing above 1.5 % GO showed nonlinear loss of constants values *ε*′ and *ε*″, while these constants in the case of PU without admixtures had values 5 and below 0.1 and changed with frequency insignificantly [6]. In general, electric properties of polyurethanes are important, when they are applied as corrosion protection coatings [7], electrochromic actual devices [,^8^] and nonlinear electric or piezoelectric sensors [9]; therefore, at present there are many research tasks conducted on improvement of polyurethane layers’ electric properties. The presented paper is consistent with the research on developing new ecological water-thinnable PU acrylic topcoats, which would allow obtaining thin polymeric films electrically conductive as so-called intelligent materials with the electrical properties which would change influenced by frequency and intensity of the applied electric field.

## Experimental

### Materials

The research included the polymer films prepared from polyurethane cationomer produced from polyurethane cationomers with added graphene. Polyurethane cationomer as polymer matrix for 0–2 wt.% graphene was synthesized in the reaction of MDI, polycaprolactone diol, and *N*-metyldiethanoloamine. In order to obtain nanocomposites, graphene was previously noncovalent functionalized in THF in the field of ultrasound. The reference films were prepared by covering poly(tetrafluoroethylene) (PTFE) or glass plates by the polyurethane cationomers water dispersions. Next, the films were placed in a vacuum drier, at 80 °C, for over 6 h, and the process of film formation was completed by exposure to ambient air during 10 days. In this way, polymer films containing respectively 0 (PU-0 sample), 0.1 (PU-0.1), 0.5 (PU-0.5), 1 (PU-1), 1.5 (PU-1.5), or 2 wt.% (PU-2) of the graphene were received [1].

### Volume resistivity

The measurements of volume resistivity were taken in accordance with the fopllowing standards:

- IEC 93:1980 Methods of test for volume resistivity and surface resistivity of solid electrical insulating materials.
- ASTM D 257-99 Standard test methods for dc resistance or conductance of insulating materials.

**Fig. 1:**
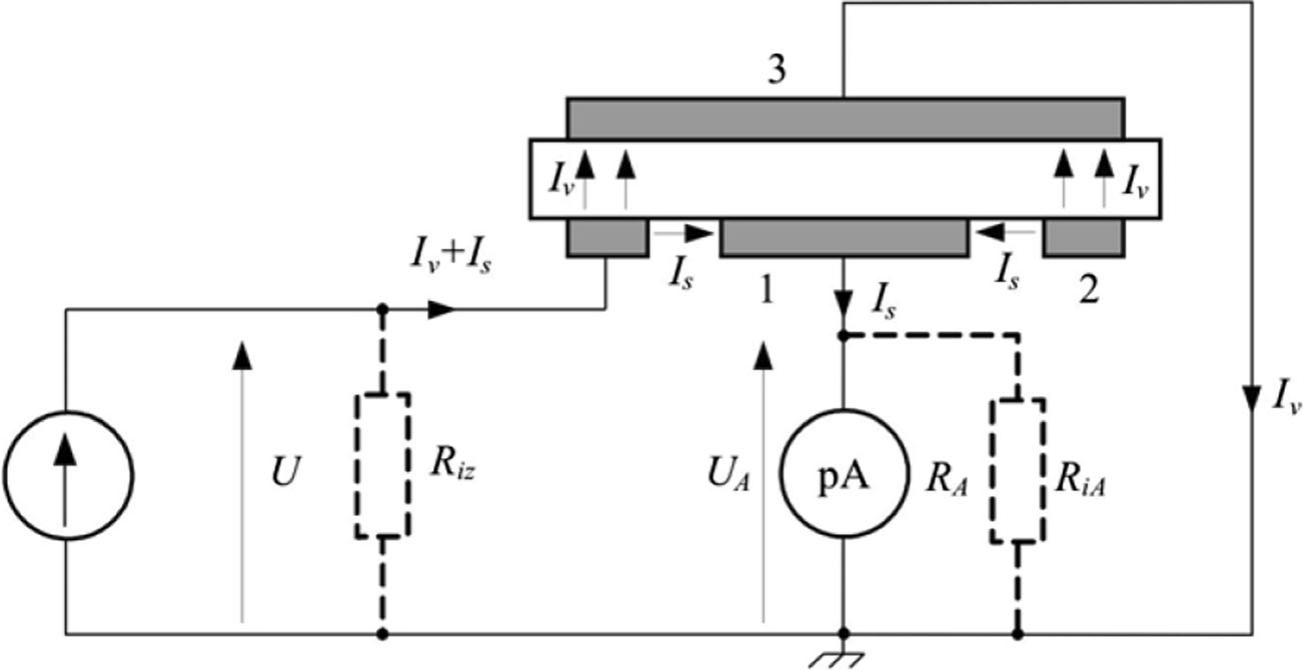
Electric circuit used for measurement of surface resistivity in the three electrode setup. Electrodes: (*1*) measuring, (*2*) voltage, (*3*) grounded

There was a measuring setup used based on three electrodes, presented in Fig. 1. Measurements were taken with Electrometer Keithley 6517A, with electrodes 8009.

### Dielectric properties

The measurements of dielectric properties were taken in the system containing three electrodes in temperature of 20 °C in the frequency range of 10^−4^–5 × 10^3^ Hz. The relaxation processes and the basic dielectric parameters were defined on the basis of frequency domain spectroscopy (FDS). The system DIRANA of OMICRON was used to conduct measurements. The software Origin v.8.0 and WinFit of Novocontrol was used to present and analyze the results of the measurements. The generic complex equation Havriliak–Negami (H-N) equation was applied to set basic relaxation functions’ parameters in frequency, which described the changes of dielectric permittivity in frequency function [10, 11]:

1 $$ \varepsilon \left(\omega \right)=-j{\left(\frac{\sigma_0}{\varepsilon_0\omega}\right)}^N+{\displaystyle \sum_{k=1}^2\left(\frac{\varDelta {\varepsilon}_k}{{\left(1+{\left(j\omega {\tau}_k\right)}^{\alpha_k}\right)}^{\beta_k}}+{\varepsilon}_{\infty k}\right)} $$

where

*k*: is the total number of partial (*k* = 1 for low-frequency component HN1 and *k* = 2 for high-frequency component HN2);
*N*: is the coefficient of conductivity character;
$$ \varDelta \varepsilon ={\varepsilon}_0-{\varepsilon}_{\infty } $$
is the polarizability, the difference between static permittivity (low frequency) (*ε*_0_) and optical high-frequency permeability $\left({\varepsilon}_{\infty}\right)$;
Δ*ε*_*k*_: is the polarizability;
*τ*_*k*_: is the relaxation time;
$$ \omega =2\pi \times f $$
is circular frequency;
*f*: is vibration frequency;
*α*, *β*: are the constants of the equation H-N; and
*σ*_0_: is the parameter of AC conductivity.

## Results and discussion

The most important properties of the obtained films were described in the paper [1] and they are presented in Table 1. It is relevant because the electrical properties presented in this paper are interpreted in the context of a chemical structure and the physical properties of the obtained materials.

**Table 1:** Physicomechanical properties of the synthesized PU films [1]

Sample no.	Graphene content in the PU film, wt.%	Glass transition of soft segments, °C T_g1 inflection point_	Glass transition of hard segments, °C T_g2 inflection point_	Thermal properties (by TG analysis)			Mechanical properties			Surface free energy, 0.001 J m^−2^	The statistical parameters of the surface roughness by AFM height^a^ sensor			
		By conventional DSC		*T*_5%_	*T*_max_	Ash,%	*σ*_*r*_, MPa	*ε*_*r*_, %	*E*, MPa	*γ*_*S*_	Surface area, μm^2^	*R*_*a*_, nm	*R*_*q*_, nm	*R*_max_, nm
PU-0	0	−49.7	23.9	115	497	4.67	5.51	145	162	45.3	2500	28.4	35.2	361
PU-0.1	0.10	−49.4	30.9	115	495	5.40	68.07	471	334	42.3	2500	65.7	94.4	887
PU-0.5	0.50	–	–	116	499	6.57	5.82	130	85	41.4	–	–	–	–
PU-1.0	1.0	−45.2	32.3	172	495	6.80	38.65	508	267	38.7	–	–	–	–
PU-1.5	1.5	−47.9	37.3	180	491	7.50	13.17	462	194	39.4	–	–	–	–
PU-2	2.0	−43.4	38.6	182	496	11.24	58.35	509	383	38.4	2500	15.4	32.0	657

^a^Surface area: the total area of examined sample surface (the three-dimensioned area of a given region expressed as the sum of the area of all the triangles formed by three adjacent data points)

*R*_a_* (*mean roughness*) the mean value of the surface relative to the center place, *R*_q_** (*R*_*ms*_) the standard deviation of the *Z* values within the given area, *R*_max_*** (*max height*) the difference in height between the highest and lowest points on the surface relative to the mean plane, *Mean* the average of all *Z* values within the enclosed area

**Scheme 1:**
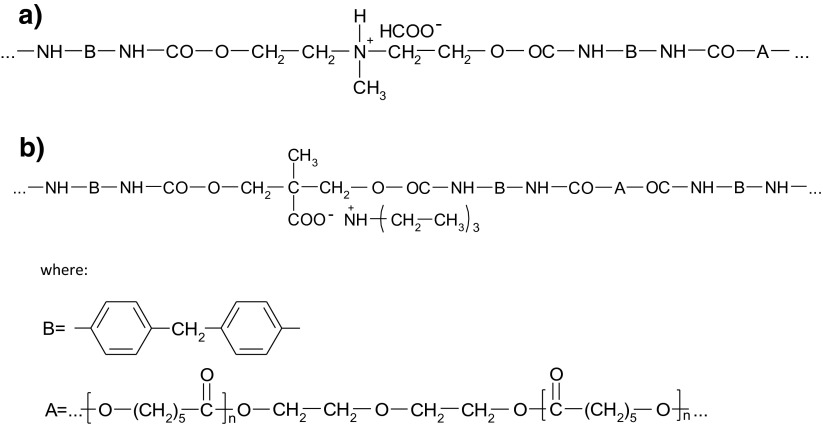
Ionic structure in polyurethane cationomer (**a**) and anionomer (**b**)

Regarding data presented Table 2, it can be inferred that the volume resistivity of the tested polymer matrix is approximately 6 × 10^8^ Ω m. It is the amount lower than thermoplastic polyurethane called Tradename WHT–1570 made from polyester and MDI as a standard elastomer with *T*_*g*_ = −40 °C, its volume resistivity is more than 10^11^ Ω m [4]. The previously tested polymer film which was obtained from anionomer polyurethane synthesized from MDI, polyoxypropylene diol (*M* = 450) and 2,2-bis(hydroxymethyl)propionic acid (DMPA) had the volume resistivity of 10^11^ Ω m [12]. The volume resistivity of the currently tested film is much smaller even without the graphene admixture. The chemical structure of the polymer and its phase construction have significant impact on decrease of resistivity. The tested PU is segmented polar polyurethane cationomer. In the hard segments of the PU, there are ionic groups: alkylammonium cations in the main chain and formate anions acting as counter anion that is presented on Scheme 1a. The phase construction of cationomer is crucial; it allows to form the elastomer film. The elastomer is constructed of flexible segments (*T*_*g*1_ = −49.7 °C) and hard segments (*T*_*g*2_ = −23.9 °C), that give it good mechanical properties and considerable polarity with high surface energy of 45.3 × 10^−3^ J m^−2^. In the chain of anionomers analyzed in the paper [12], there were clusters of ion, but contrary to the cationomers, the anions were less mobile since they originated from DMPA acid built in the main chain (Scheme 1b). It can be inferred that conductivity in the analyzed cationomer is definitely ionic, and the formate anions have the pivotal role in transporting of charges. It should be emphasized that 2 % concentration graphene results in the decrease in the volume resistivity of three orders of magnitude 6.0 × 10^8^ Ω m for the pure material to 3.4 × 10^5^ Ω m. The surface of approximately 1 % of the graphene admixture where the sudden decrease of resistivity is probably caused by the exceed of percolation threshold is noticeable. It should be noticed that a little smaller amount of percolation threshold (0.5–1 %) was observed in polyurethane composites modified by multi-walled carbon nanotubes (MWCNT), where the significant decrease in resistivity from 2.5 × 10^12^ Ω m to 5–6 orders of magnitude was observed [4].

**Table 2:** The volume resistivity results of polymer films

Sample no.	Multiplicity of the measurement	The measuring voltage *U* = 100 V	
		Volume resistivity ρ_s_ [Ω m]	The value average value, ρ_s_ [Ω m]
PU-2	1	2.6 × 10^5^	3.4 × 10^5^
	2	1.0 × 10^5^	
	3	6.6 × 10^5^	
PU-1.5	1	7.6 × 10^6^	8.2 × 10^6^
	2	8.0 × 10^6^	
	3	9.0 × 10^6^	
PU-1.0	1	8.8 × 10^7^	8.9 × 10^7^
	2	8.9 × 10^7^	
	3	9.0 × 10^7^	
PU-0.5	1	1.5 × 10^8^	1.4 × 10^8^
	2	1.2 × 10^8^	
PU-0.1	1	2.3 × 10^8^	2.4 × 10^8^
	2	2.2 × 10^8^	
	3	2.8 × 10^8^	
PU-0	1	6.4 × 10^8^	6.0 × 10^8^
	2	6.3 × 10^8^	
	3	5.4 × 10^8^

Significantly more cross-linked structure polyurethane/hyperbranched poly(urea-uretane) doped the similar amount of MWCNTs did not show any significant changes in resistivity [13]. It can be claimed that apart from the content of graphene, the line structure of the cationomer’s chain is the crucial factor helping decrease resistivity.

**Fig. 2:**
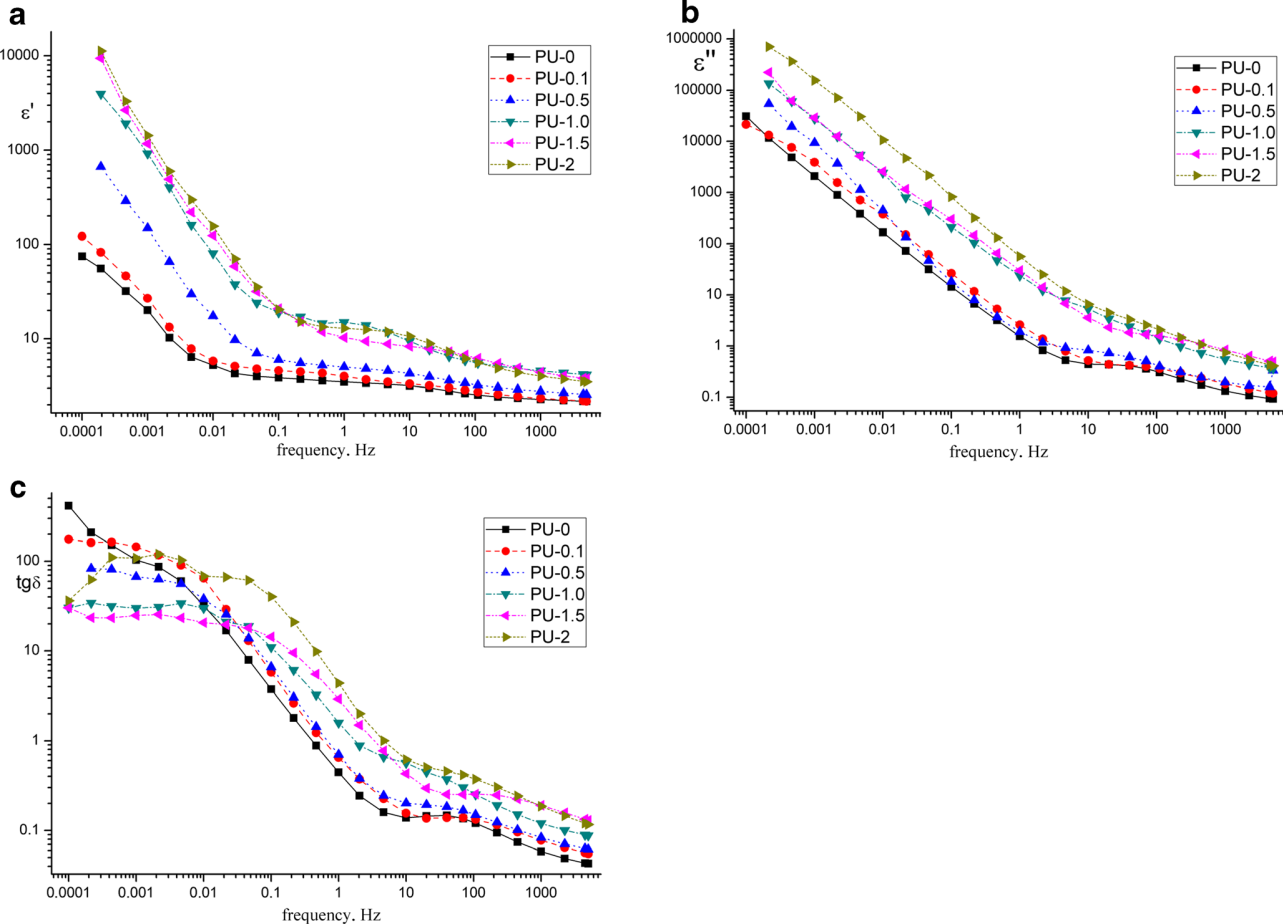
Changes of the real part of permittivity *ε*′ (**a**), imaginary part of permittivity *ε*″ (**b**) and of dielectric losses coefficient tanδ (**c**) in the frequency domain

Having analyzed the obtained FDS characteristics (Fig. 2a, b), it results to the increase of the graphene concentration causing the increase in the real value of the part of electric permittivity *ε*′ (capacitance) and imaginary part of electric permittivity *ε*″ (dielectric losses) over the whole measurement range of frequency. The obtained parameters *ε*′ and *ε*″ for the polyurethane cationomers which have not been doped are approximately 1–2 orders of magnitude higher than the ones registered in the case of the polyurethane anionomers [12] as well as doped polyurethanes 0.5–3 % oxide graphene [6] that confirms the higher polarity and ionic activity within the chains of cationomers and is compatible with the significant increase of conductivity of nanocomposites by graphene, which was noncovalent functionalized in THF which was observed.

On the basis of changes of dielectric loss factor tgδ, it has been stated that the occurrence of those two relaxation processes (Fig. 2c). They are characterized by the local maximums on the graph tgδ in the low-frequency range from 0.1 Hz and in the range HF over 10 Hz. The high-frequency process is probably related to the so-called effects *α*-relaxation occurring in the area of hard urethane segments, which is especially visible in the DSC and DMTA research in the temperature *T*_*g*2_ in the extent of 25–40 °C. While the low-frequency relaxation *β* (type Johari–Goldstein [14]) is related to the processes occurring in the flexible polyester segments and it is registered to be below 0 °C in the DSC measurements. In the researched cationomers the range is *T*_*g*1_ = −40 to –50 °C. The relaxation Maxwell–Wagner [15] occurring in the border of phases composite surface of the samples and the air cannot be excluded; however, it is less likely regarding its roughness of surface of the researched polymer films (Table 1).

The parameters of FDS characteristics, estimated with H-N equation for tested polyurethane films are given in Table 3. The quantitative analysis of the dielectric response of samples with addition of graphene was performed with H-N Eq. (1), taking into consideration AC conductivity parameter *σ*_0,_ and there was an analysis of observed relaxation processes conducted in tested samples.

**Table 3:** FDS parameters determined by the Havriliak–Negami Eq. (1) (for *k* = 1 and *k* = 2) for the examined polyurethane films

Sample no.	*σ*_0_, Ω^−1^ m^−1^		For H-N 1 equation		For H-N 2 equation	
PU-0	*σ*_0_	1.01e−12	Δ*ε*	8.8	Δ*ε*	1.5
			*τ*	579	*τ*	5.4e−3
	*N*	1	*ε*_∞_	1.07	*ε*_∞_	1.45
			*α*	0.82	*α*	0.59
			*β*	1	*β*	1
PU-0.1	*σ*_0_	1.59e−12	Δ*ε*	141	Δ*ε*	2.2
			*τ*	616	*τ*	10.6e−3
	*N*	1	*ε*_∞_	1.07	*ε*_∞_	1.43
			*α*	0.84	*α*	0.5
			*β*	1	*β*	1
PU-0.5	*σ*_0_	2.49e−12	Δ*ε*	1.3	Δ*ε*	2.2
			*τ*	643	*τ*	4.2e−3
	*N*	1	*ε*_∞_	1.30	*ε*_∞_	1.44
			α	0.86	*α*	0.65
			β	1	*β*	1
PU-1	*σ*_0_	5.03e−12	Δ*ε*	5038	Δ*ε*	8.5
			*τ*	716	*τ*	13e−3
	*N*	1	*ε*_∞_	1.71	*ε*_∞_	1.45
			*α*	0.891	*α*	0.46
			*β*	1	*β*	1
PU-1.5	*σ*_0_	1.19e−11	Δ*ε*	1e5	Δ*ε*	5.5
			*τ*	3700	*τ*	1.4e−3
	N	1	*ε*_∞_	2,16	*ε*_∞_	1.45
			*α*	0.91	*α*	0.6
			*β*	1	*β*	1
PU-2	*σ*_0_	4.46e−11	Δ*ε*	1e5	Δ*ε*	7.14
			*τ*	3770	*τ*	3.3e−3
	*N*	1	*ε*_∞_	2.26	*ε*_∞_	1.37
			*α*	0.912	*α*	0.78
			*β*	1	*β*	1

**Fig. 3:**
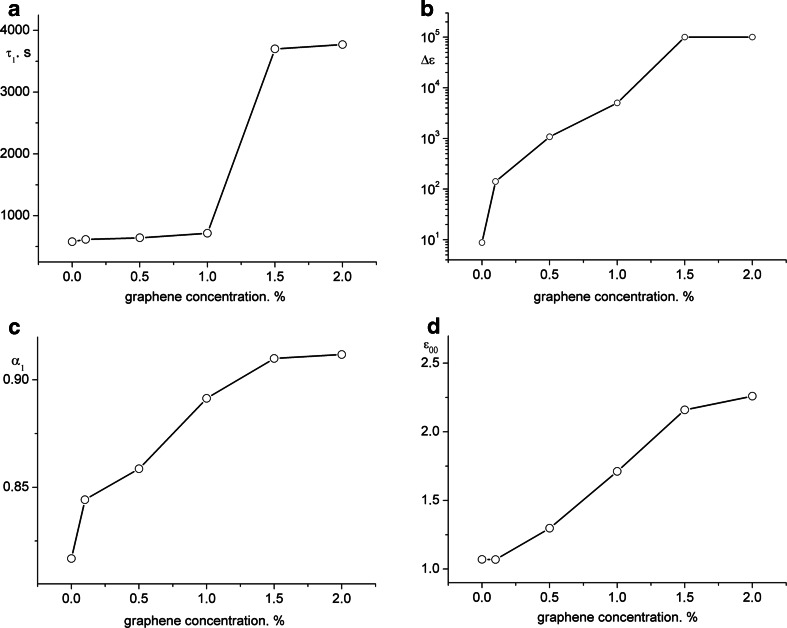
Changes of time constant *τ*_1_ (**a**), polarizability Δ*ε* (**b**), *α*_1_ parameter (**c**), and optical permeability parameter *ε*_∞_ (**d**) of low frequency relaxation process taking place in tested cationomers with various contents of graphene

The parameter *N* = 1 occurring in the equation H-N allows to conclude that in the tested films the ionic conductivity prevails. The increase of the graphene concentration in the range from 0.1 to 2 % results in logarithmic increase of AC conductivity parameter *σ*_0_ (conductivity) from 1e−12 Ω^−1^ m^−1^ for the undoped material to 4.5e−11 Ω^−1^ m^−1^ for 2 % of admixture. It is the process related to the increase of the concentration of ions and the change of structure as well. The admixture of graphene significantly influences the structural relaxation of the material which is observed in prolongation of the relaxation time and the rapid increase of the parameter value *τ* when amount of admixture exceeds 1 % is visible (Fig. 3a). This effect is probably related to exceeding percolation threshold, which in the case of other tested carbon nanostructures (MWCNT, fullerene) or expanded graphene may take place at low concentration of value of 1 % [4, 13]. The above-mentioned conclusions are confirmed by recorded changes of polarizability of tested samples, which after exceeding percolation threshold, had parameter Δ*ε* practically set on the maximal value (Fig. 3b). In Fig. 3c, changes of *α*_1_ parameter are presented (low-frequency relaxation process) for various concentration of graphene in tested polyurethane cationomers. The increase of admixture amount causes the parameter *α*_1_ to reach the value of 1, which confirms Debye character of relaxation (Fig. 3d), and with very high value of polarizability and high time constant, it allows to suppose that ions present in polymer chains are highly mobile when they are exposed to external electric field.

## Conclusions

The research showed that polymer film made from waterborne cationomer polyurethane is characterized with lower volume resistivity, on the level 6.0 × 10^8^ Ω m than in the cases of layers made from polyurethane and polyurethane anionomer produced from the same diisocyanates and similar in the structure polyols. Also, these cationomers have higher values of the real part of permittivity *ε*′ (capacity) and imaginary part of permittivity *ε*″ (dielectric losses), which can be explained by the high polarity and the phase structure. In the relaxation phenomena in the tested material, there are probably dominant interactions of the *dipole*–*dipol*e type; therefore, a strong frequency dispersion of constants *ε*′ and *ε*″ especially in the range of 10^−4^–10^−1^ Hz and significant increase of relaxation time (with the increase of graphene contents) especially at low frequency are observed. The admixture of graphene in the amount 0.5–2 wt.% causes the increase of conductivity and percolation threshold is set on the level of approx. 1 wt.%. The values of constants *ε*′ and *ε*″ clearly grow with the increase of graphene amount with the similar shape of frequency dispersion curves as for PU without admixture. There has been the large decrease of PU resistivity in the presence of graphene, which was noncovalent functionalization in THF solvent in the field of ultrasound gives a significant advantage if compared to nonconducting polyurethane compounds obtained with oxide graphene.
